# Supplementary material for: Spatial dilemmas of diffusible public goods
Source: eLife. 2013 Dec 17;2:e01169. doi: 10.7554/eLife.01169 (PMC3865686; doi:10.7554/eLife.01169)
Supplement: Supplementary file 1. — DOI: http://dx.doi.org/10.7554/eLife.01169.008 [file elife01169s001.pdf]

Supplementary File 1 for "Spatial dilemmas of diffusible public goods" Mathematical proofs

Benjamin Allen[1,2], Jeff Gore[3], and Martin A. Nowak[2,4]

[1] *Department of Mathematics, Emmanuel College, Boston, Massachusetts 02115, USA*
[2] *Program for Evolutionary Dynamics, Harvard University, Cambridge, Massachusetts 02138, USA*
[3] *Department of Physics, Massachusetts Institute of Technology, Cambridge, Massachusetts 02139, USA*
[4] *Department of Mathematics, Department of Organismic and Evolutionary Biology, Harvard University, Cambridge, Massachusetts 02138, USA*

December 6, 2013

1 Model

We begin by reviewing the model described in the main text. Spatial population structure is represented by a weighted graph G of N nodes, with each cell occupying a node. The edge weight from cell i to cell j is denoted e_{ij}. G is required to have bi-transitive symmetry, which means that for every pair of nodes $i, j \in G$, there is a graph isomorphism of G interchanging i and j (Taylor et al., 2007).

In any given state of the evolutionary process, the stationary public goods concentration at node $i \in G$ is denoted ψ_i. We discuss how ψ_i is determined from the diffusion process in later sections. The fecundity of cell i is given by $F_i = 1 + \delta(b\psi_i - cs_i)$, where b and c represent benefit and cost of the public good, respectively, and $s_i = 0, 1$ identifies the type (cooperator or defector, respectively) of cell i. Here we have introduced a new parameter, $\delta > 0$,

to quantify the strength of selection. This differs from the notation used in the main text, in that δb and δc as used here correspond to just b and c, respectively, in the main text. This change allows for cleaner derivations but has no effect on our results.

We primarily consider two update rules, *birth-death* and *death-birth*, as introduced by Ohtsuki et al. (2006). For birth-death, first a cell is chosen to reproduce, with probability proportional to fecundity. The offspring displaces a neighbor of the parent, chosen at random with probability proportional to edge weight. For death-birth, first a cell is chosen to die with uniform probability. Then a neighbor is chosen to produce offspring to fill the new vacancy, with probability proportional to fecundity times edge weight. In the case of a cycle, we will consider an additional update rule, *shift* (Allen and Nowak, 2012), defined in Section 6.2.3.

2 Results for arbitrary diffusion processes

The first results we derive are valid for any diffusion process that respects the symmetries of the graph G. (This includes the case that the diffusion is described by a graph G' that is different from the reproduction graph G.) The physical diffusion process described in the main text is a special case, which we explore starting in Section 3.

An arbitrary diffusion process is defined by a collection of quantities $\{\phi_{ij} \geq 0\}_{i,j \in G}$, where ϕ_{ij} represents the fraction of non-decayed public good that, if produced by cell i, would be absorbed by cell j under this process. We require that the ϕ_{ij} satisfy

(i) $\sum_{j \in G} \phi_{ij} = 1$ for each $i \in G$, and

(ii) $\phi_{T(i)\,T(j)} = \phi_{ij}$ for any isomorphism T of G.

Condition (i) asserts that these fractions add to one, while condition (ii) ensures that symmetries in the graph topology are reflected in the sharing of public goods. For any given state of the evolutionary process, the stationary concentration at node $i \in G$ is then defined as $\psi_i = \sum_{j \in G} s_j \phi_{ji}$. The physical diffusion process described in the main text is a special case, which we explore starting in Section 3.

As in the main text, we define $\phi_0 = \phi_{ii}$ as the average amount of public good retained by its producer, and $\phi_1 = \sum_{h \in G} e_{hi} \phi_{hi}$ as the weighted average

amount received by the producer's neighbors. By symmetry, it does not mater which node is chosen as i in these definitions.

2.1 Recurrence relations for identity-by-descent

We study spatial assortment of types using identity-by-descent (IBD) probabilities (Rousset and Billiard, 2000; Rousset, 2004; Taylor et al., 2007; Allen et al., 2012). For this analysis we consider a "dummy" mutation rate u. Since we are not interested in the effects of mutation, we will eventually take the limit $u \to 0$. This allows us to analyze the spatial assortment of types under neutral drift.

Two cells i and j are identical by descent if no mutation separates either of them from their common ancestor. We let q_{ij} denote the probability that i and j are identical by descent in the stationary distribution over states of the evolutionary process.

By the arguments of Taylor et al. (2007) and Allen et al. (2012), the stationary IBD probabilities q_{ij} for $i \neq j$ satisfy

$$\begin{aligned} q_{ij} &= (1-u)\sum_{h\in G} e_{ih} q_{hj} \\ &= (1-u)\sum_{h\in G} q_{ih} e_{hj}. \end{aligned}$$

Obviously we have $q_{ii} = 1$. We can combine the cases $i \neq j$ and $i = j$ using Kronecker delta notation:

$$\begin{aligned} q_{ij} &= \delta_{ij}(1-(1-u)q_1) + (1-u)\sum_{h\in G} e_{ih} q_{hj} \\ &= \delta_{ij}(1-(1-u)q_1) + (1-u)\sum_{h\in G} q_{ih} e_{hj}. \end{aligned}$$

Above, $q_1 = \sum_{h\in G} e_{ih} q_{ih}$ is the weighted average IBD probability of an individual to its neighbors, and δ_{ij} equals one if $i = j$ and zero otherwise. Rearranging, we get

$$\begin{aligned} \sum_{h\in G} e_{ih} q_{hj} &= \frac{q_{ij} - \delta_{ij}(1 - q_1 + u q_1)}{1-u} \\ &= \begin{cases} q_1 & i = j \\ q_{ij}/(1-u) & i \neq j. \end{cases} \end{aligned} \tag{1}$$

2.2 Fecundity under weak selection

To analyze the limit of weak selection, we consider a "focal individual", denoted $\bullet$. By the required symmetry of the graph, it does not matter which individual is chosen.

We proceed following the approach of Allen et al. (2012). Given that $\bullet$ is a cooperator, the probability that some other individual $i \in G$ is a cooperator, under neutral drift, can be obtained from the IBD probability $q_{\bullet i}$:

$$\langle s_i \rangle_{\substack{\delta=0\\ s_\bullet=1}} = \frac{1+q_{\bullet i}}{2}.$$

Above, the notation $\langle\ \rangle_{\substack{\delta=0\\ s_\bullet=1}}$ refers to the expectation of a quantity under the stationary distribution of states of the neutral ($\delta = 0$) evolutionary process, conditioned on the focal individual being a cooperator ($s_\bullet = 1$); see Allen et al. (2012).

The expected stationary concentration of public good at any node i, conditioned on $\bullet$ being a cooperator, can then be calculated as

$$\begin{aligned}
\langle \psi_i \rangle_{\substack{\delta=0\\ s_\bullet=1}} &= \sum_{j\in G} \langle s_j \phi_{ji} \rangle_{\substack{\delta=0\\ s_\bullet=1}} \\
&= \sum_{j\in G} \frac{1+q_{\bullet j}}{2}\phi_{ij} \\
&= \frac{1}{2} + \frac{1}{2}\sum_{j\in G} q_{\bullet j}\phi_{ij}.
\end{aligned}$$

The expected fecundity of individual i, conditioned on $\bullet$ being a cooperator, is therefore given by

$$\begin{aligned}
\langle F_i \rangle_{\substack{\delta=0\\ s_\bullet=1}} &= 1 + \delta \big\langle b\psi_i - cs_i \big\rangle_{\substack{\delta=0\\ s_\bullet=1}} \\
&= 1 + \delta \left[\frac{b-c}{2} + \frac{1}{2}\left(b\sum_{j\in G} q_{\bullet j}\phi_{ij} - cq_{\bullet i} \right) \right]. \qquad (2)
\end{aligned}$$

2.3 Weak-selection conditions for cooperation

We now obtain conditions under which cooperation is favored under weak selection, depending on the update rule, population size, and diffusion process. We let $b_\bullet$ (resp., $d_\bullet$) denote the probability that the focal individual

reproduces (resp., dies) in the current time step. Nowak et al. (2010) and Allen et al. (2012) showed that cooperation is favored under weak selection and rare mutation if and only if

$$\left\langle \frac{\partial(b_\bullet - d_\bullet)}{\partial\delta} \right\rangle_{\substack{\delta=0\\ s_\bullet=1}} > 0. \tag{3}$$

In words, cooperation is favored under weak selection if the difference between the birth rate and the death rate of cooperators increases as selection strength is increased from zero.

2.3.1 Birth-Death

We first consider birth-death updating. The birth and death probabilities, respectively, of the focal individual under this update rule can be written as

$$b_\bullet = \frac{F_\bullet}{\sum_{j\in G} F_j} \qquad d_\bullet = \frac{\sum_{i\in G} e_{i\bullet} F_i}{\sum_{j\in G} F_j}.$$

Using the fact that $F_i = 1$ for each $i \in G$ when $\delta = 0$, we obtain

$$\left\langle \frac{\partial(b_\bullet - d_\bullet)}{\partial\delta} \right\rangle_{\substack{\delta=0\\ s_\bullet=1}} = \frac{1}{N}\left(\left\langle \frac{\partial F_\bullet}{\partial\delta} \right\rangle_{\substack{\delta=0\\ s_\bullet=1}} - \sum_{i\in G} e_{i\bullet} \left\langle \frac{\partial F_i}{\partial\delta} \right\rangle_{\substack{\delta=0\\ s_\bullet=1}} \right). \tag{4}$$

According to condition (3), cooperation is favored if and only if the above quantity is positive. From (2) we have

$$\left\langle \frac{\partial F_i}{\partial\delta} \right\rangle_{\substack{\delta=0\\ s_\bullet=1}} = \frac{b-c}{2} + \frac{1}{2}\left(b\sum_{j\in G} q_{\bullet j}\phi_{ij} - cq_{\bullet i} \right),$$

which we substitute into (4) and (3) to obtain the success condition

$$b\left(\sum_{j\in G} q_{\bullet j}\phi_{\bullet j} - \sum_{i\in G}\sum_{j\in G} e_{\bullet i} q_{\bullet j}\phi_{ij} \right) - c(1-q_1) > 0. \tag{5}$$

To simplify the middle sum in (5), we first note that, by symmetry, the focal individual $\bullet$ can be replaced by an average over all individuals $h \in G$:

$$\sum_{i,j} e_{\bullet i} q_{\bullet j}\phi_{ij} = \frac{1}{N}\sum_{i,j,h} e_{hi} q_{hj}\phi_{ij}.$$

We then simplify by applying (1) as follows:

$$\begin{aligned}\frac{1}{N}\sum_{i,j,h} e_{hi}q_{hj}\phi_{ij} &= \frac{1}{N}\sum_{i,j}\phi_{ij}\sum_{h} e_{hi}q_{hj}\\ &= \frac{1}{N(1-u)}\sum_{i,j}\phi_{ij}\left[q_{ij}-\delta_{ij}(1-q_1+uq_1)\right]\\ &= \frac{1}{N(1-u)}\left[\sum_{i,j}\phi_{ij}q_{ij}-\sum_{i}\phi_{ii}(1-q_1+uq_1)\right]\\ &= \frac{1}{1-u}\sum_{i}\phi_{\bullet i}q_{\bullet i}-\phi_0(1-q_1+uq_1).\end{aligned}$$

Substituting this result in condition (5) yields

$$b\left[-\frac{u}{1-u}\sum_{i\in G} q_{\bullet i}\phi_{\bullet i}+\phi_0(1-q_1+uq_1)\right]-c(1-q_1)>0.$$

We now substitute the relation $q_1 = 1-(N-1)u+\mathcal{O}(u^2)$ obtained by Taylor et al. (2007), yielding:

$$bu\left(-\sum_{i\in G} q_{\bullet i}\phi_{\bullet i}+N\phi_0\right)-cu(N-1)+\mathcal{O}(u^2)>0.$$

Finally, we divide by u and let $u\to 0$, noting that $q_{\bullet h}\to 1$ for each $h\in G$ in this limit. This gives

$$b\left(-1+N\phi_0\right)-c(N-1)>0$$

We conclude that cooperation is favored iff

$$\frac{b}{c}>\frac{N-1}{N\phi_0-1}.$$

In the large-population limit ($N\to\infty$), this becomes $b/c>1/\phi_0$, or $b\phi_0>c$.

2.3.2 Death-Birth

In the case of DB updating, birth and death probabilities are given by

$$b_\bullet = \frac{1}{N}\sum_{i\in G}\frac{e_{\bullet i}F_\bullet}{\sum_{j\in G} e_{ji}F_j}, \qquad d_\bullet = \frac{1}{N}.$$

Again using the fact that $F_i = 1$ for each i when $\delta = 0$, we have

$$\left\langle \frac{\partial(b_\bullet - d_\bullet)}{\partial \delta} \right\rangle_{\substack{\delta=0\\ s_\bullet=1}} = \frac{1}{N}\left(\left\langle \frac{\partial F_\bullet}{\partial \delta} \right\rangle_{\substack{\delta=0\\ s_\bullet=1}} - \sum_{i\in G}\sum_{j\in G} e_{\bullet i} e_{ji} \left\langle \frac{\partial F_j}{\partial \delta} \right\rangle_{\substack{\delta=0\\ s_\bullet=1}} \right).$$

Substituting from (2) and invoking condition (3), we obtain that cooperators are favored if and only if

$$b\left(\sum_{h\in G} q_{\bullet h}\phi_{\bullet h} - \sum_{i\in G}\sum_{j\in G}\sum_{h\in G} e_{\bullet i} e_{ji} q_{\bullet h}\phi_{hj} \right) - c\left(1 - \sum_{i\in G}\sum_{j\in G} e_{\bullet i} e_{ji} q_{\bullet j}\right) > 0. \quad (6)$$

We first simplify the sum in the coefficient of c using the recurrence relations (1):

$$\begin{aligned}
\sum_{i\in G}\sum_{j\in G} e_{\bullet i}\, e_{ji}\, q_{\bullet j} &= \sum_{i\in G} e_{\bullet i} \sum_{j\in G} e_{ji} q_{\bullet j} \\
&= \frac{1}{1-u}\sum_{i\in G} e_{\bullet i}\big(q_{\bullet i} - \delta_{\bullet i}(1 - q_1 + u q_1)\big) \\
&= \frac{1}{1-u}\sum_{i\in G} e_{\bullet i} q_{\bullet i} \\
&= \frac{q_1}{1-u}.
\end{aligned}$$

From the second to the third line above we used the fact that $e_{\bullet\bullet} = 0$ (no self-replacement). The last equality comes from the definition of q_1 as the weighted average IBD probability of a cell to its neighbors.

We now simplify the sum in the b coefficient by repeated use of (1):

$$\begin{aligned}
\sum_{i\in G}\sum_{j\in G}\sum_{h\in G} e_{\bullet i} e_{ji} q_{\bullet h}\phi_{hj} &= \frac{1}{N}\sum_{\ell,i,j,h} e_{\ell i} e_{ji} q_{\ell h}\phi_{hj} \\
&= \frac{1}{N}\sum_{i,j,h} e_{ji}\phi_{hj}\sum_{\ell} e_{\ell i} q_{\ell h} \\
&= \frac{1}{N(1-u)}\sum_{i,j,h} e_{ji}\phi_{hj}\big(q_{ih} - \delta_{ih}(1-q_1+uq_1)\big) \\
&= \frac{1}{N(1-u)}\left(\sum_{i,j,h} e_{ji}\phi_{hj} q_{ih} - \sum_{i,j} e_{ji}\phi_{ij}(1-q_1+uq_1)\big)\right) \\
&= \frac{1}{1-u}\left(\frac{1}{N}\sum_{j,h}\phi_{hj}\sum_{i} e_{ji} q_{ih} - \phi_1(1-q_1+uq_1)\big)\right) \\
&= \frac{1}{N(1-u)^2}\sum_{j,h}\phi_{hj}\big(q_{jh} - \delta_{jh}(1-q_1+uq_1)\big) \\
&\qquad - \frac{\phi_1(1-q_1+uq_1)\big)}{1-u} \\
&= \frac{1}{N(1-u)^2}\left(\sum_{j,h}\phi_{hj} q_{jh} - \sum_{j}\phi_{jj}(1-q_1+uq_1)\big)\right) \\
&\qquad - \frac{\phi_1(1-q_1+uq_1)\big)}{1-u} \\
&= \frac{1}{(1-u)^2}\sum_{j}\phi_{\bullet j} q_{j\bullet} - \frac{\phi_0(1-q_1+uq_1)}{(1-u)^2} \\
&\qquad - \frac{\phi_1(1-q_1+uq_1)\big)}{1-u}.
\end{aligned}$$

Substituting into (6), we obtain the condition

$$\begin{aligned}
b\left[\left(1-\frac{1}{(1-u)^2}\right)\sum_{h\in G} q_{\bullet h}\phi_{\bullet h} + \frac{1-q_1+uq_1}{(1-u)^2}\phi_0 + \frac{1-q_1+uq_1}{1-u}\phi_1\right] \\
- c\left(1-\frac{q_1}{1-u}\right) > 0.
\end{aligned}$$

Substituting $q_1 = 1 - (N-1)u + \mathcal{O}(u^2)$ (Taylor et al., 2007) yields:

$$bu \left(-2 \sum_{h \in G} q_{\bullet h} \phi_{\bullet h} + N\phi_0 + N\phi_1 \right) - cu(N-2) + \mathcal{O}(u^2) > 0.$$

Finally, we divide by u and let $u \to 0$, noting that $q_{\bullet h} \to 1$ for each $h \in G$ in this limit. This gives

$$b\left(-2 + N\phi_0 - N\phi_1\right) - c(N-2) > 0.$$

We conclude that cooperation is favored if and only if

$$\frac{b}{c} > \frac{N-2}{N(\phi_0 + \phi_1) - 2}.$$

In the large-population limit ($N \to \infty$), this becomes

$$b/c > \frac{1}{\phi_0 + \phi_1},$$

which is condition (2) of the main text.

3 Recurrence relations for ϕ_{ij}

We now consider the specific diffusion process described in equation (1) of the main text. For this process, the fractions ϕ_{ij} of amount of public good absorbed by cell j from that produced by cell i satisfy the recurrence relations

$$\begin{aligned} \phi_{ij} &= \frac{\delta_{ij}}{1+\lambda} + \frac{\lambda}{1+\lambda} \sum_{h \in G} e_{hi} \phi_{hj} \\ &= \frac{\delta_{ij}}{1+\lambda} + \frac{\lambda}{1+\lambda} \sum_{h \in G} e_{hj} \phi_{ih}. \end{aligned} \tag{7}$$

These recurrence relations are the same as those for the stationary concentrations ψ_j in a state where only i is a cooperator.

In particular, setting $i = j$, we have the following relation between the amount $\phi_0 = \phi_{ii}$ retained by the producer and the weighted average amount $\phi_1 = \sum_{h \in G} e_{hi} \phi_{hi}$ that goes to the producer's neighbors:

$$\phi_0 = \frac{1}{1+\lambda} + \frac{\lambda}{1+\lambda} \phi_1. \tag{8}$$

The fractions ϕ_{ij} can be expressed in terms of generating functions that characterize random walks on G. The probabilities of each possible step in these random walks are given by the edge weights. Let $p_{ij}^{(n)}$ denote the probability that a random walk starting at i will be at node j after n steps. Let $r_{ij}^{(n)}$ denote the probability that a random walk starting at i will be at node j after n steps, without having visited j in any previous step. We define the *Green's generating function*

$$\mathcal{G}_{ij}(z) = \sum_{n=0}^{\infty} p_{ij}^{(n)} z^n,$$

and the *first visit generating function*

$$\mathcal{F}_{ij}(z) = \sum_{n=0}^{\infty} r_{ij}^{(n)} z^n.$$

The recurrence relations (7) then imply

$$\phi_{ij} = \frac{1}{1+\lambda} \mathcal{G}_{ij} \left(\frac{\lambda}{1+\lambda} \right). \tag{9}$$

The generating functions $\mathcal{F}_{ij}(z)$ and $\mathcal{G}_{ij}(z)$ are related by identity

$$\mathcal{G}_{ij}(z) = \mathcal{G}_{jj}(z) \mathcal{F}_{ij}(z). \tag{10}$$

This reflects the fact that a random walk that starts at i and ends at j must visit j for the first time at some point, and subsequently return to j.

4 Sufficient and asymptotically necessary conditions for success

Using the results of the previous section, we now derive conditions for cooperation to be favored under the physical diffusion process on any graph. The conditions we obtain are expressed in terms of the diffusion parameter λ and the effective graph degree κ. These conditions are *sufficient* for cooperation to be favored, and they also become necessary as the diffusion rate λ approaches zero.

4.1 Birth-Death

We have established the condition $b/c > \phi_0^{-1}$ for cooperator success under birth-death updating in the limits of weak selection and large population size. From equation (8) we have

$$\phi_0^{-1} = \frac{1+\lambda}{1+\lambda\phi_1}. \tag{11}$$

It immediately follows that $\phi_0^{-1} < 1+\lambda$. The condition $b/c > 1+\lambda$ therefore implies $b/c > \phi_0^{-1}$, and is consequently sufficient for cooperation to be favored.

We now show that this condition is asymptotically necessary in the limit $\lambda \to 0$. To begin, we use the generating function formula (9) for ϕ_{ij} to obtain the asymptotic behavior of ϕ_1 as $\lambda \to 0$:

$$\begin{aligned}
\phi_1 &= \sum_{j\in G} e_{ij}\phi_{ij} \\
&= \frac{1}{1+\lambda}\sum_{j\in G} e_{ij}\mathcal{G}_{ij}\left(\frac{\lambda}{1+\lambda}\right) \\
&= \frac{1}{1+\lambda}\sum_{j\in G} e_{ij}^2\frac{\lambda}{1+\lambda} + \mathcal{O}(\lambda^2) \\
&= \frac{\lambda}{(1+\lambda)^2\kappa} + \mathcal{O}(\lambda^2) \\
&= \frac{\lambda}{\kappa} + \mathcal{O}(\lambda^2).
\end{aligned} \tag{12}$$

Combining (11) and (12) yields

$$\phi_0^{-1} = 1+\lambda+\mathcal{O}(\lambda^2). \tag{13}$$

Thus the condition $b/c > 1+\lambda$ is asymptotically necessary as $\lambda \to 0$.

4.2 Death-Birth

We have established the condition $b/c > (\phi_0+\phi_1)^{-1}$ for the success of cooperation under death-birth updating and weak selection. From (12) and (13) we have

$$(\phi_0+\phi_1)^{-1} = 1+\frac{\kappa-1}{\kappa}\lambda+\mathcal{O}(\lambda^2/\kappa).$$

Thus the condition $b/c > 1+\frac{\kappa-1}{\kappa}\lambda$ is asymptotically necessary for cooperation to be favored as $\lambda \to 0$. The following lemma now shows that this condition is also sufficient, for any λ.

Lemma 1. *$(\phi_1 + \phi_0)^{-1} < 1 + \lambda(\kappa - 1)/\kappa$.*

Proof. For ease of notation, we introduce the quantity $\eta = \lambda/(1 + \lambda)$. We then have

$$\phi_1 = (1 - \eta) \sum_{j\in G} e_{ij}\mathcal{G}_{ij}(\eta). \tag{14}$$

We recall that $e_{ii} = 0$. Furthermore, for $i \neq j$, we have

$$\mathcal{G}_{ij}(\eta) \geq \eta e_{ij} + \eta^2 \sum_{h\in G} e_{ih}^2 \mathcal{G}_{ij}(\eta). \tag{15}$$

The first term on the right-hand side above represents one-step walks from i to j, while the second represents walks that step first to a node h, then return to i on the next step before eventually terminating at j. The inequality reflects the fact that the walks described above are only a subset of all finite walks starting at i and terminating at j.

Substituting (14) into (15) yields

$$\phi_1 \geq (1 - \eta) \sum_{j\in G} e_{ij}^2\eta + \sum_{h\in G} e_{ih}^2\eta^2\phi_1 = \frac{\eta(1 - \eta)}{\kappa} + \frac{\eta^2}{\kappa}\phi_1.$$

This gives the inequality

$$\phi_1 \geq \frac{\eta(1 - \eta)}{\kappa - \eta^2}. \tag{16}$$

Relation (8) can be expressed in terms of η as

$$\phi_0 = 1 - \eta + \eta\phi_1. \tag{17}$$

Combining (16) and (17), we obtain

$$\phi_0 + \phi_1 \geq 1 - \eta + \frac{(1 + \eta)\eta(1 - \eta)}{\kappa - \eta^2}. \tag{18}$$

The statement of the lemma is equivalent to

$$\phi_0 + \phi_1 > \frac{1 - \eta}{1 + \eta(\kappa - 2)}.$$

By (18), it suffices to show that
$$1-\eta+\frac{(1+\eta)\eta(1-\eta)}{\kappa-\eta^2} > \frac{1-\eta}{1+\eta(\kappa-2)}.$$
Noting that $\eta < 1$, we factor out $1-\eta$ and rearrange, yielding the equivalent condition
$$1+\frac{\eta(1+\eta)}{\kappa-\eta^2} - \frac{1}{1+\eta(\kappa-2)} > 0.$$
Factoring the left-hand side above yields
$$\frac{\eta(\kappa-1)(\eta+\kappa-1)}{(\kappa-\eta^2)\big(1+\eta(\kappa-2)\big)} > 0.$$
This claim now follows from $\kappa > 1$ and $0 < \eta < 1$. □

5 Calcuation of ϕ_0 for various graph structures

Having obtained general conditions for the success of cooperation under weak selection, we now turn to specific graph structures. In this section we calculate the fraction ϕ_0 of public goods retained by the cooperator under the physical diffusion process on various well-known graphs.

5.1 Well-mixed

A well-mixed population is represented by a complete graph, with all edge weightings equal to $1/(N-1)$. On such graphs, by symmetry, an equal amount of public good is absorbed by each cell other than the producer; that is, $\phi_{ij} = \phi_1$ for each $i \neq j$. We therefore have
$$\phi_0 + (N-1)\phi_1 = 1.$$
Combining with (8) and solving the resulting system of equations for ϕ_0 yields
$$\phi_0 = \frac{N+\lambda-1}{N+N\lambda-1}.$$
In the large-population limit ($N \to \infty$), this becomes
$$\phi_0 = \frac{1}{1+\lambda},$$
as stated in Table 1 of the main text.

5.2 Lattices

For n-dimensional square lattices of side length ℓ, with periodic boundary conditions, Montroll and Weiss (1965) derived expressions for the generating function $\mathcal{G}_{ij}(z)$. Combining their solutions with (9) yields the following expression for ϕ_{ij}:

$$\phi_{ij} = \phi_{\mathbf{x}} \equiv \frac{1}{N} \sum_{\mathbf{y} \in G} \frac{\exp\left(-2\pi i\, \mathbf{x} \cdot \mathbf{y}\right)}{1 + \lambda - \frac{\lambda}{n} \sum_{i=1}^{n} \cos(2\pi\, y_i/\ell)}. \tag{19}$$

In particular,

$$\phi_0 = \frac{1}{N} \sum_{\mathbf{y} \in G} \frac{1}{1 + \lambda - \frac{\lambda}{n} \sum_{i=1}^{n} \cos(2\pi\, y_i/\ell)}.$$

For infinite lattices, we take the limit $\ell \to \infty$ and the sum becomes an integral:

$$\phi_0 = \frac{1}{(2\pi)^n} \int_{-\pi}^{\pi} \cdots \int_{-\pi}^{\pi} \frac{d\theta_1 \ldots d\theta_n}{1 + \lambda - \frac{\lambda}{n} \sum_{i=1}^{n} \cos\theta_i}.$$

In particular, for an infinite one-dimensional lattice ($n = 1$), we have

$$\phi_0 = \frac{1}{\sqrt{1 + 2\lambda}}.$$

For a cycle of size N ($n = 1$ and $\ell = N$), we have

$$\phi_0 = \frac{1}{\sqrt{1 + 2\lambda}} \frac{1 + \gamma^N}{1 - \gamma^N}, \qquad \gamma = \frac{1 + \lambda - \sqrt{1 + 2\lambda}}{\lambda}.$$

For an infinite two-dimensional lattice ($n = 2$), a result of Shore and Tyler (1993) implies

$$\phi_0 = \frac{1}{\operatorname{agm}(1, 1 + 2\lambda)}.$$

5.3 Bethe lattices

The first-visit generating function on a Bethe lattice (infinite Cayley tree) is given by (e.g. Allen et al., 2012)

$$\mathcal{F}_{ij}(z) = \left(\frac{k - \sqrt{k^2 - 4z^2(k-1)}}{2z(k-1)} \right)^{d(i,j)}.$$

Equations (9) and (10) imply

$$\frac{\phi_{ij}}{\phi_{ii}} = \mathcal{F}_{ij}\left(\frac{\lambda}{1+\lambda}\right).$$

In particular, choosing a neighboring pair for i and j,

$$\frac{\phi_1}{\phi_0} = \frac{k(1+\lambda) - \sqrt{k^2(1+\lambda)^2 - 4\lambda^2(k-1)}}{2\lambda(k-1)}.$$

Combining with (8) and solving for ϕ_0 yields

$$\phi_0 = \frac{\sqrt{(k-2)^2(1+\lambda)^2 + 4(k-1)(1+2\lambda)} - (k-2)(1+\lambda)}{2(1+2\lambda)}.$$

6 Results for arbitrary selection strength

We now derive results for well-mixed and cycle-structured popultions that are valid for any selection strength δ in the required range $0 < \delta \leq 1/(c-b)$ (recall that $\delta > 1/(c-b)$ results in negative reproductive rates and is therefore not allowed in our model). We make use of the fact that, for well-mixed and cycle-structured populations, the current state of the Markov chain representing evolution can be represented—without any loss of information—by the number k of cooperators present in this state. In well-mixed populations, this property arises from the interchangeability of spatial positions. In cycles, this property arises from the fact that, in the limit of low mutation, the only states that arise are those in which cooperators and defectors form two separate clusters of like types (Ohtsuki and Nowak, 2006).

In these cases, the fixation probabilities of cooperators and defectors, ρ_C and ρ_D respectively, are related by the following identity (Nowak et al., 2004; Taylor et al., 2004):

$$\frac{\rho_C}{\rho_D} = \prod_{k=1}^{N} \frac{p_k^+}{p_k^-}. \tag{20}$$

Above, p_k^+ (resp., p_k^-) is the probability of a transition to one more (resp., less) cooperator, given that there are currently k cooperators.

6.1 Well-mixed

We recall that a well-mixed population is represented by a complete graph. In a state with k cooperators, each cooperator is connected to $k-1$ cooperators and $N-k$ defectors, and each defector is connected to k cooperators and $N-k-1$ defectors.

We recall from the main text that the stationary concentrations of public good at each node satisfy the recurrence relations

$$\begin{cases} \psi_i = \dfrac{1}{1+\lambda} + \dfrac{\lambda}{1+\lambda} \displaystyle\sum_{j \in G} e_{ji} \psi_j & s_i = 1 \\ \psi_i = \dfrac{\lambda}{1+\lambda} \displaystyle\sum_{j \in G} e_{ji} \psi_j & s_i = 0. \end{cases} \tag{21}$$

For the well-mixed population, each cooperator experiences the same stationary concentration, which we call ψ_C, and each defector experiences the same stationary concentration, which we call ψ_D. From (21) and the complete graph structure, we see that ψ_C and ψ_D are related by

$$\begin{aligned} \psi_C &= \frac{1}{1+\lambda} + \frac{\lambda}{1+\lambda} \frac{(k-1)\psi_C + (N-k)\psi_D}{N-1} \\ \psi_D &= \frac{\lambda}{1+\lambda} \frac{k\psi_C + (N-k-1)\psi_D}{N-1}. \end{aligned}$$

Subtracting, we obtain

$$\psi_C - \psi_D = \frac{1}{1+\lambda} - \frac{\lambda}{1+\lambda} \frac{\psi_C - \psi_D}{N-1}.$$

Solving for $\psi_C - \psi_D$ yields

$$\psi_C - \psi_D = \frac{N-1}{N-1+N\lambda}. \tag{22}$$

We recall that fecundities (reproductive rates) are given in this model by

$$F_i = \begin{cases} 1 + \delta(b\psi_i - c) & s_i = 1 \\ 1 + \delta b \psi_i & s_i = 0. \end{cases}$$

Again by symmetry, all cooperators have the same fecundity, F_C, and all defectors have the same fecundity, F_D. Combining with (22), we see that

$$F_C > F_D \iff b\frac{N-1}{N-1+N\lambda} > c. \tag{23}$$

Interestingly, this condition does not involve the number k of cooperators; thus cooperators are either favored in every state or disfavored in every state. It is elementary to show that, for either birth-death or death-birth updating on a complete graph, $p_k^+ > p_k^-$ if and only if $F_C > F_D$. We therefore conclude from (23) that cooperators are favored in a well-mixed population of size N if and only if

$$\frac{b}{c} > 1 + \frac{N}{N-1}\lambda.$$

In the large-population limit ($N \to \infty$), this becomes $b/c > 1 + \lambda$.

6.2 Cycles

For $i = 0 \dots N-1$,

$$\phi_i = \frac{1}{\sqrt{1+2\lambda}} \frac{\gamma^i + \gamma^{N-i}}{1-\gamma^N}, \qquad \gamma = \frac{1+\lambda-\sqrt{1+2\lambda}}{\lambda}.$$

Consider an arbitrary state of the evolutionary process, with k cooperators present, $0 \leq k \leq N$. Label the cells so that cells $i = 0, \dots, k-1$ are cooperators and cells $k, \dots, N-1$ are defectors. Then

$$\psi_i = \frac{1}{\sqrt{1+2\lambda}(1-\gamma^N)} \sum_{j=0}^{k-1} \left(\gamma^{|i-j|} + \gamma^{N-|i-j|} \right).$$

We now examine each update rule separately.

6.2.1 Birth-Death

In birth-death updating on a cycle, the probabilities of increase or decrease in the numbers of cooperators are determined solely by the fecundities of cells at the boundary between cooperators and defectors. In the above labeling scheme, the relevant cells are the cooperators indexed 0 and $k-1$ (which have the same fecundity as each other by symmetry), and the defectors indexed k and $N-1$ (which again have the same fecundity as each other). In particular, the ratio of the probabilities of increase versus decrease in the number of cooperators is given by

$$\frac{p_k^+}{p_k^-} = \frac{F_0}{F_{N-1}} = \frac{1+\delta(b\psi_0 - c)}{1+\delta b \psi_{N-1}}.$$

Above, ψ_0 and ψ_{N-1} are the amounts of public good received by cooperators and defectors, respectively, at the boundary between cooperator and defector clusters. For cooperators, this amount can be calculated as

$$\begin{aligned}\psi_0 &= \frac{1}{\sqrt{1+2\lambda}(1-\gamma^N)} \sum_{j=0}^{k-1} (\gamma^j + \gamma^{N-j}) \\ &= \frac{1}{\sqrt{1+2\lambda}} \frac{(1-\gamma^k)(1+\gamma^{N+1-k})}{(1-\gamma)(1-\gamma^N)}.\end{aligned} \tag{24}$$

For defectors, we obtain

$$\begin{aligned}\psi_{N-1} &= \frac{1}{\sqrt{1+2\lambda}(1-\gamma^N)} \sum_{j=0}^{k-1} (\gamma^{j+1} + \gamma^{N-j-1}) \\ &= \frac{1}{\sqrt{1+2\lambda}} \frac{\gamma(1-\gamma^k)(1+\gamma^{N-1-k})}{(1-\gamma)(1-\gamma^N)}.\end{aligned} \tag{25}$$

To prove that the condition $b/c > \sqrt{1+2\lambda}$ is necessary and sufficient for cooperator success in the limit $N \to \infty$, we define the function

$$f_N(x) = \log\left(\frac{p^+_{\lfloor Nx \rfloor}}{p^-_{\lfloor Nx \rfloor}}\right).$$

Above, $\lfloor y \rfloor$ denotes the greatest integer less than or equal to y (also known as the "floor function" of y). Then, using eq. (20), we have

$$\frac{\rho_C}{\rho_D} = \exp\left[(N-1)\int_{1/N}^{1} f_N(x)\, dx\right].$$

We now analyze the limit $N \to \infty$. For each fixed x, $0 < x < 1$,

$$\lim_{N\to\infty} f_N(x) = \log\left(\frac{1+\delta(b\psi_C^* - c)}{1+\delta b\psi_D^*}\right),$$

with ψ_C^* and ψ_D^* denoting the limiting amounts of public good received by cooperators and defectors, respectively, on the boundary between large clusters:

$$\begin{aligned}\psi_C^* &= \lim_{\substack{N\to\infty \\ k=\lfloor Nx \rfloor}} \psi_0 = \frac{1}{\sqrt{1+2\lambda}} \frac{1}{1-\gamma} \\ \psi_D^* &= \lim_{\substack{N\to\infty \\ k=\lfloor Nx \rfloor}} \psi_{N-1} = \frac{1}{\sqrt{1+2\lambda}} \frac{\gamma}{1-\gamma}.\end{aligned} \tag{26}$$

We observe also that $f_N(x)$ is bounded, for each N and x, by $\log\left[1+\delta(b-c)\right]$ above and by $\log\left[(1-\delta c)/(1+\delta b)\right]$ below. The lower bound is finite and real since we have assumed $\delta < 1/c$. Thus by the Lebesgue Dominated Convergence Theorem,

$$\begin{aligned}
\lim_{N\to\infty} \frac{\rho_C}{\rho_D} &= \lim_{N\to\infty} \exp\left((N-1)\int_{1/N}^{1} f_N(x)\,dx\right)\\
&= \lim_{N\to\infty} \exp\left[(N-1)\int_{1/N}^{1} \log\left(\frac{1+\delta(b\psi_C^* - c)}{1+\delta b\psi_D^*}\right)\,dx\right]\\
&= \lim_{N\to\infty} \exp\left[(N-1)\log\left(\frac{1+\delta(b\psi_C^* - c)}{1+\delta b\psi_D^*}\right)\right]\\
&= \begin{cases} \infty & b\psi_C^* - c > \psi_D^* \\ 1 & b\psi_C^* - c = \psi_D^* \\ 0 & b\psi_C^* - c < \psi_D^*. \end{cases}
\end{aligned}$$

We conclude that $\rho_C > \rho_D$ in the limit $N \to \infty$, for any $0 < \delta < 1/c$, if and only if $b\psi_C^* - c > b\psi_D^*$. Substituting from (26), we obtain $b/c > \sqrt{1+2\lambda}$ as a necessary and sufficient condition for cooperator success.

6.2.2 Death-Birth

For death-birth updating, the ratio of probabilities of increase versus decrease of the current number k of cooperators, $1 \le k \le N-1$, is

$$\begin{aligned}
\frac{p_k^+}{p_k^-} &= \frac{F_0}{F_0 + F_{N-2}} \frac{F_1 + F_{N-1}}{F_{N-1}}\\
&= \frac{1 + F_1/F_{N-1}}{1 + F_{N-2}/F_0}.
\end{aligned}$$

Following the argument of the previous section, let

$$f_N(x) = \log\left(\frac{p_{\lfloor Nx \rfloor}^+}{p_{\lfloor Nx \rfloor}^-}\right).$$

We then have

$$\frac{\rho_C}{\rho_D} = \exp\left[(N-1)\int_{1/N}^{1} f_N(x)\,dx\right].$$

For each fixed x, $0 < x < 1$,

$$\lim_{N\to\infty} f_N(x) = \log\left(\frac{1+\dfrac{1+\delta(b\psi_C^{**}-c)}{1+\delta b\psi_D^{*}}}{1+\dfrac{1+\delta(b\psi_C^{*}-c)}{1+\delta b\psi_D^{**}}}\right).$$

Above, ψ_C^* and ψ_D^* are defined as in (26), while ψ_C^{**} and ψ_D^{**} denote the limiting amounts of public good received by cooperators and defectors, respectively, one position removed from the boundary between large clusters. For cooperators, we calculate

$$\begin{aligned}\psi_C^{**} &= \lim_{\substack{N\to\infty\\ k=\lfloor Nx\rfloor}} \psi_1 \\ &= \lim_{N\to\infty} \frac{1}{\sqrt{1+2\lambda}(1-\gamma^N)} \sum_{j=0}^{\lfloor Nx\rfloor-1} \left(\gamma^{|j-1|}+\gamma^{N-|j-1|}\right) \\ &= \frac{1}{\sqrt{1+2\lambda}}\left(\gamma+\sum_{j=1}^{\infty}\gamma^{j-1}\right) \\ &= \frac{1}{\sqrt{1+2\lambda}}\left(\gamma+\frac{1}{1-\gamma}\right).\end{aligned} \tag{27}$$

For defectors, we obtain

$$\begin{aligned}\psi_D^{**} &= \lim_{\substack{N\to\infty\\ k=\lfloor Nx\rfloor}} \psi_{N-2} \\ &= \lim_{N\to\infty} \frac{1}{\sqrt{1+2\lambda}(1-\gamma^N)} \sum_{j=0}^{\lfloor Nx\rfloor-1} \left(\gamma^{j+2}+\gamma^{N-j-2}\right) \\ &= \frac{1}{\sqrt{1+2\lambda}}\sum_{j=0}^{\infty}\gamma^{j+2} \\ &= \frac{1}{\sqrt{1+2\lambda}}\frac{\gamma^2}{1-\gamma}.\end{aligned} \tag{28}$$

We also have that for each $N \geq 2$, and $1/N \leq x < 1$, $f_N(x)$ is bounded below by $-\log(2+\delta b)$ and above by $\log[2+\delta(b-c)]$. By the Lebesgue

Dominated Convergence Theorem,

$$\begin{aligned}
\lim_{N\to\infty} \frac{\rho_C}{\rho_D} &= \lim_{N\to\infty} \exp\left[(N-1)\int_{1/N}^{1} f_N(x)\, dx\right] \\
&= \lim_{N\to\infty} \exp\left[(N-1)\int_{1/N}^{1} \log\left(\frac{1+\dfrac{1+\delta(b\psi_C^{**}-c)}{1+\delta b\psi_D^{*}}}{1+\dfrac{1+\delta(b\psi_C^{*}-c)}{1+\delta b\psi_D^{**}}}\right) dx\right] \\
&= \lim_{N\to\infty} \exp\left[(N-1)\log\left(\frac{1+\dfrac{1+\delta(b\psi_C^{**}-c)}{1+\delta b\psi_D^{*}}}{1+\dfrac{1+\delta(b\psi_C^{*}-c)}{1+\delta b\psi_D^{**}}}\right)\right].
\end{aligned}$$

Thus cooperators are favored in the large-population limit if and only if the ratio in parentheses above is greater than one. We observe that the numerator is greater than one if and only if $\psi_C^{**} - \psi_D^{*} > c/b$, while the denominator is less than one if and only if $\psi_C^{*} - \psi_D^{**} > c/b$. From eqs. (26), (27), and (28), we have

$$\psi_C^{**} - \psi_D^{*} = \psi_C^{*} - \psi_D^{**} = \frac{1+\gamma}{\sqrt{1+2\lambda}}.$$

Thus cooperation is favored if and only if

$$\frac{b}{c} > \frac{\sqrt{1+2\lambda}}{1+\gamma} = \frac{1+\sqrt{1+2\lambda}}{2}.$$

6.2.3 Shift

Finally, we consider a third update rule on the cycle, *shift* (Allen and Nowak, 2012). Under this rule, at each time step, one cell is selected to reproduce with probability proportional to fitness. Simultaneously, another cell is selected to die with uniform probability. The new daughter cell appears adjacent its parent, and the remaining cells shift along the cycle to accommodate the new cell and fill the vacancy.

For shift updating, the ratio of increase to decrease in cooperators can be expressed as (Allen and Nowak, 2012)

$$\frac{p_k^+}{p_k^-} = \frac{\bar{F}_C}{\bar{F}_D}.$$

Above, $\bar{F}_C$ and $\bar{F}_D$ denote the average fecundities of cooperators and defectors, respectively.

As above, we define

$$f_N(x) = \log\left(\frac{p^+_{\lfloor Nx \rfloor}}{p^-_{\lfloor Nx \rfloor}}\right).$$

For each fixed x, it is straightforward to show that, as $N \to \infty$ with $k = \lfloor Nx \rfloor$, $\bar{F}_C$ approches $1 + \delta(b - c)$ while $\bar{F}_D$ approaches 1. (That is, the concentration of public good near the average cooperator converges to one, while the concentration of public good near the average defector converges to 0 as $N \to \infty$.) We therefore have

$$\lim_{N\to\infty} f_N(x) = \log\big[1 + \delta(b - c)\big].$$

We also have that $f_N(x)$ is bounded, for each N and x, by $\log\big[1 + \delta(b - c)\big]$ above and by $\log\big[(1 - \delta c)/(1 + \delta b)\big]$ below. By the arguments used for the other update rules, we conclude that cooperation is favored in the limit $N \to \infty$, for any valid selection strength, if and only if $b > c$.

References

Taylor, P. D., Day, T., & Wild, G. (2007) *Nature* **447**, 469–472.

Ohtsuki, H., Hauert, C., Lieberman, E., & Nowak, M. A. (2006) *Nature* **441**, 502–505.

Allen, B. & Nowak, M. A. (2012) *Journal of Theoretical Biology* **311**, 28 – 39. http://www.sciencedirect.com/science/article/pii/S0022519312003402.

Rousset, F. & Billiard, S. (2000) *Journal of Evolutionary Biology* **13**, 814–825.

Rousset, F. (2004) *Genetic structure and selection in subdivided populations* (Princeton University Press, Princeton).

Allen, B., Traulsen, A., Tarnita, C. E., & Nowak, M. A. (2012) *Journal of Theoretical Biology* **299**, 97–105. http://www.sciencedirect.com/science/article/pii/S0022519311001809.

Nowak, M. A., Tarnita, C. E., & Wilson, E. O. (2010) *Nature* **466**, 1057–1062.

Montroll, E. & Weiss, G. (1965) *Journal of Mathematical Physics* **6**, 167.

Shore, T. & Tyler, D. (1993) *American Mathematical Monthly* 144–149.

Ohtsuki, H. & Nowak, M. A. (2006) *Proceedings of the Royal Society B: Biological Sciences* **273**, 2249–2256. http://rspb.royalsocietypublishing.org/content/273/1598/2249.abstract.

Nowak, M. A., Sasaki, A., Taylor, C., & Fudenberg, D. (2004) *Nature* **428**, 646–650.

Taylor, C., Fudenberg, D., Sasaki, A., & Nowak, M. (2004) *Bulletin of Mathematical Biology* **66**, 1621–1644. http://dx.doi.org/10.1016/j.bulm.2004.03.004. 10.1016/j.bulm.2004.03.004.
